# Supplementary material for: Swine intestinal segment perfusion model for the evaluation of nutrients bioaccessibility
Source: PLoS One. 2023 Apr 14;18(4):e0283825. doi: 10.1371/journal.pone.0283825 (PMC10104296; doi:10.1371/journal.pone.0283825)

PLOS ONE

Swine intestinal segment perfusion model for the evaluation of nutrients bioaccessibility

--Manuscript Draft--

**Manuscript Number:**	PONE-D-22-35014
**Article Type:**	Research Article
**Full Title:**	Swine intestinal segment perfusion model for the evaluation of nutrients bioaccessibility
**Short Title:**	Swine intestinal segment perfusion model for the evaluation of nutrients bioaccessibility
**Corresponding Author:**	Luciana Rossi<br>University of Milan: Universita degli Studi di Milano<br>Lodi, ITALY
**Keywords:**	duodenum; intestinal segment; nutrient bioaccessibility; absorption; swine; alternative models to experimental animals
**Abstract:**	The nutrition science requires more science-based evidences for the development of effective functional diets. To reduce animals for experimental purposes innovative reliable and informative models, simulating the complex intestinal physiology, are needed. The aim of this study was to develop a swine duodenum segment perfusion model for the evaluation of nutrient bioaccessibility and functionality among time. At slaughterhouse, one sow intestine was harvested from 100 kg following Maastricht criteria for cardiac death organ donation (CDC) for transplantation purposes. Duodenum tract was isolated and perfused in sub-normothermic conditions with heterologous blood after cold ischemia induction. Duodenum segment perfusion model was maintained under controlled pressure conditions through extracorporeal circulation for 3 hours. Blood samples from extracorporeal circulation and luminal content samples were collected at regular intervals for the evaluation of glucose concentration by glucometer, minerals (Na+, Ca2+, Mg2+, K+) by ICP-OES, lactate-dehydrogenase and nitrite oxide by spectrophotometric methods. The macroscopic observation showed peristaltic activity caused by intrinsic nerves. Glycemia decreased over time (from 44.00±1.20 mg/dL to 27.50±0.41; $p < 0.01$), suggesting glucose utilization by the tissue confirming the organ viability in line with histological examinations. At the end of the experimental period, intestinal mineral concentrations resulted lower than their level in blood plasma suggesting their bioaccessibility ($p < 0.001$). A progressive increase of LDH concentration over time was observed in the luminal content probably related to a loss of viability (from 0.32±0.02 to 1.36±0.02 OD; $p < 0.05$) confirmed by histological findings that revealed a de-epithelization of the distal portion of duodenum. Isolated swine duodenum perfusion model satisfied the criteria for studying bioaccessibility of nutrients, offering a variety of experimental possibilities in line with 3Rs principle.
**Order of Authors:**	Matteo Dell'Anno
	Fabio Acocella
	Pietro Riccaboni
	Camilla Recordati
	Elisabetta Bongiorno
	Luciana Rossi
**Additional Information:**	
**Question**	**Response**
**Financial Disclosure**<br><br>Enter a financial disclosure statement that describes the sources of funding for the	This research was funded by Lombardy Region (funding number: POR FESR 2014–2020_BANDO Call HUB Ricerca e Innovazione_D.G.R. NR 727 del 5 November 2018) within the project MIND FoodS Hub (Milano Innovation District Food System Hub): Innovative concept for the eco-intensification of agricultural production and for

work included in this submission. Review the submission guidelines for detailed requirements. View published research articles from *PLOS ONE* for specific examples.<br><br>This statement is required for submission and **will appear in the published article** if the submission is accepted. Please make sure it is accurate.<br><br>**Unfunded studies**<br>Enter: *The author(s) received no specific funding for this work.*<br><br>**Funded studies**<br>Enter a statement with the following details:<br>• Initials of the authors who received each award<br>• Grant numbers awarded to each author<br>• The full name of each funder<br>• URL of each funder website<br>• Did the sponsors or funders play any role in the study design, data collection and analysis, decision to publish, or preparation of the manuscript?<br>• **NO** - Include this sentence at the end of your statement: *The funders had no role in study design, data collection and analysis, decision to publish, or preparation of the manuscript.*<br>• **YES** - Specify the role(s) played.<br><br>* typeset	the promotion of dietary patterns for human health and longevity through the creation in MIND of a digital Food System Hub. The funder has no role in defining the study, in data collection, management, analysis or interpretation of the data, nor in the writing and submitting of manuscripts resulting from this study.
**Competing Interests**<br><br>Use the instructions below to enter a competing interest statement for this submission. On behalf of all authors, disclose any competing interests that could be perceived to bias this work—acknowledging all financial support and any other relevant financial or non-financial competing interests.<br><br>This statement is required for submission and **will appear in the published article** if the submission is accepted. Please make sure it is accurate and that any funding sources listed in your Funding Information	The authors declare that the research was conducted in the absence of any commercial or financial relationships that could be construed as a potential conflict of interest.

later in the submission form are also declared in your Financial Disclosure statement.<br><br>View published research articles from *PLOS ONE* for specific examples.<br><br>**NO authors have competing interests**<br><br>Enter: *The authors have declared that no competing interests exist.*<br><br>**Authors with competing interests**<br><br>Enter competing interest details beginning with this statement:<br><br>*I have read the journal's policy and the authors of this manuscript have the following competing interests: [insert competing interests here]*<br><br>* typeset	
**Ethics Statement**<br><br>Enter an ethics statement for this submission. This statement is required if the study involved:<br><br>• Human participants<br>• Human specimens or tissue<br>• Vertebrate animals or cephalopods<br>• Vertebrate embryos or tissues<br>• Field research<br><br>Write "N/A" if the submission does not require an ethics statement.<br><br>General guidance is provided below. Consult the submission guidelines for detailed instructions. **Make sure that all information entered here is included in the Methods section of the manuscript.**	N/A

**Format for specific study types**<br><br>**Human Subject Research (involving human participants and/or tissue)**<br>• Give the name of the institutional review board or ethics committee that approved the study<br>• Include the approval number and/or a statement indicating approval of this research<br>• Indicate the form of consent obtained (written/oral) or the reason that consent was not obtained (e.g. the data were analyzed anonymously)<br><br>**Animal Research (involving vertebrate animals, embryos or tissues)**<br>• Provide the name of the Institutional Animal Care and Use Committee (IACUC) or other relevant ethics board that reviewed the study protocol, and indicate whether they approved this research or granted a formal waiver of ethical approval<br>• Include an approval number if one was obtained<br>• If the study involved *non-human primates*, add *additional details* about animal welfare and steps taken to ameliorate suffering<br>• If anesthesia, euthanasia, or any kind of animal sacrifice is part of the study, include briefly which substances and/or methods were applied<br><br>**Field Research**<br><br>Include the following details if this study involves the collection of plant, animal, or other materials from a natural setting:<br>• Field permit number<br>• Name of the institution or relevant body that granted permission	
**Data Availability**<br><br>Authors are required to make all data underlying the findings described fully available, without restriction, and from the time of publication. PLOS allows rare exceptions to address legal and ethical concerns. See the PLOS Data Policy and FAQ for detailed information.	Yes - all data are fully available without restriction

A Data Availability Statement describing where the data can be found is required at submission. Your answers to this question constitute the Data Availability Statement and **will be published in the article**, if accepted.<br><br>**Important:** Stating 'data available on request from the author' is not sufficient. If your data are only available upon request, select 'No' for the first question and explain your exceptional situation in the text box.<br><br>Do the authors confirm that all data underlying the findings described in their manuscript are fully available without restriction?	
**Describe where the data may be found in full sentences. If you are copying our sample text, replace any instances of XXX with the appropriate details.**<br><br>• If the data are **held or will be held in a public repository**, include URLs, accession numbers or DOIs. If this information will only be available after acceptance, indicate this by ticking the box below. For example: *All XXX files are available from the XXX database (accession number(s) XXX, XXX.).*<br>• If the data are all contained **within the manuscript and/or Supporting Information files**, enter the following: *All relevant data are within the manuscript and its Supporting Information files.*<br>• If neither of these applies but you are able to provide **details of access elsewhere**, with or without limitations, please do so. For example:<br><br>*Data cannot be shared publicly because of [XXX]. Data are available from the XXX Institutional Data Access / Ethics Committee (contact via XXX) for researchers who meet the criteria for access to confidential data.*<br><br>*The data underlying the results presented in the study are available from (include the name of the third party*	All relevant data are available within the manuscript

and contact information or URL).<br>• This text is appropriate if the data are owned by a third party and authors do not have permission to share the data.<br><br>* typeset	
Additional data availability information:	

Manuscript
Click here to access/download;Manuscript;Swine Intestinal Perfusion Model.docx

Swine intestinal segment perfusion model for the evaluation of nutrients bioaccessibility

Matteo Dell'Anno[1], Fabio Acocella[1], Pietro Riccaboni[1], Camilla Recordati[1], Elisabetta Bongiorno[1], Luciana Rossi[1*]

[1]Department of Veterinary Medicine and Animal Sciences - DIVAS, Università degli Studi di Milano, 26900, Lodi, Italy

* luciana.rossi@unimi.it

Abstract

The nutrition science requires more science-based evidences for the development of effective functional diets. To reduce animals for experimental purposes innovative reliable and informative models, simulating the complex intestinal physiology, are needed. The aim of this study was to develop a swine duodenum segment perfusion model for the evaluation of nutrient bioaccessibility and functionality among time. At slaughterhouse, one sow intestine was harvested from 100 kg following Maastricht criteria for cardiac death organ donation (CDC) for transplantation purposes. Duodenum tract was isolated and perfused in sub-normothermic conditions with heterologous blood after cold ischemia induction. Duodenum segment perfusion model was maintained under controlled pressure conditions through extracorporeal circulation for 3 hours. Blood samples from extracorporeal circulation and luminal content samples were collected at regular intervals for the evaluation of glucose concentration by glucometer, minerals (Na^+, Ca^{2+}, Mg^{2+}, K^+) by ICP-OES, lactate-dehydrogenase and nitrite oxide by spectrophotometric methods. The macroscopic observation showed peristaltic activity caused by intrinsic nerves. Glycemia decreased over time (from 44.00±1.20 mg/dL to 27.50±0.41; $p < 0.01$), suggesting glucose utilization by the tissue confirming the organ viability in line with histological examinations. At the end of the experimental period, intestinal mineral concentrations resulted lower than their level in blood plasma suggesting their bioaccessibility ($p < 0.001$). A progressive increase of LDH concentration over time was observed in the luminal content probably related to a loss of viability (from 0.32±0.02 to 1.36±0.02 OD; $p < 0.05$) confirmed by histological findings that revealed a de-epithelization of the distal portion of duodenum. Isolated swine duodenum perfusion model satisfied the criteria for studying bioaccessibility of nutrients, offering a variety of experimental possibilities in line with 3Rs principle.

Keywords: duodenum, intestinal segment, nutrient bioaccessibility, absorption, swine, alternative models to experimental animals

1 Introduction

Nowadays, ~~the~~ nutrition plays a pivotal role for both human and animal health, related not only to simply to satisfy nutritional requirements, it also plays a key role in the health and welfare also through its functionality (1, 2, 3, 4). The literature offers an heterogenous panorama of nutritional studies, and several studies are contradictory on the argument. The prediction of the nutritional quality of food and feed products requires more knowledge related to the individual digestibility of dietary compounds (5).

Currently, the request to reduce animals for experimental purposes is continuously growing. European policies on animal experimentation are intensely aiming to increase the protection of experimental animals, thus various alternative methods have being developed to achieve this purpose (6). Even the most advanced *in vitro* and *in silico* systems cannot fully simulate complex phenomena such as inflammation, digestion, pathologies, and metabolism. However, the introduction of innovative non-animal models is fundamental as complementary to animal experimentation (7). Thus, reliable science-based models are required in order to reduce and replace animals for experimental purposes. The gastrointestinal physiology is a complex field that involves different tissues and systems (epithelium, muscles, nerves, connective, hormones and glandule), and the use of animals for studying nutrient digestion is still considered essential.

Though remarkable results achieved with *in vitro* models, ~~the~~ organ architecture, ~~the~~ epithelium integrity and ~~the~~ nutrient absorption are not, at present, efficiently simulated. *Ex vivo* studies range in their level of complexity from simple monocultures to complex 3-D structures containing several cell types that are organized into a structure that retains (*ex vivo* tissue explants) or mimics (tissue engineered *in vitro* models) different *in vivo* elements (8). The *ex vivo* digestion models are supposed to mimic the *in vivo* processes better than the *in vitro* technology, thanks to a manufacturing process of digestive fluids, and the presence of the complete array of proteases and substances that *in vivo* concur to the digestive process (9). Several *ex vivo* studies have been conducted on pigs due to the similar characteristics of swine species in terms of similar morphology, structure, composition and enzymatic activity to humans and the low cost of this technology.

Organ explant efficiently simulate the entire animal complexity, offering the possibility to perform *ex vivo* experiments under standardized conditions and harvest several tissues from a single animal in line with the 3R principle (replace, reduce, refine). In addition, the possibility to perform more tests involving the same donor, improves the robustness of the *ex vivo* models (10). It has been demonstrated that the mucosal epithelium is extremely susceptible to ischemia (11) and although *ex vivo* models can better reproduce the complexity of the *in vivo* situation ~~rather than~~ *in vitro* models, deterioration of the explanted tissue and lack of hemodynamics can cause differences between *ex vivo* and *in vivo* data (12). The perfusion of *ex vivo* tissue allow to the conservation of organ viability and structure for a longer period compared to classic *ex vivo* models (13). Recent studies, showed encouraging results of swine intestinal perfused model suggesting it as a cost-effective, practical and reliable strategy for the study of intestinal physiology, pharmacology and transplantation (14). In this scenario, the aim of this study was to develop and assess an innovative swine intestinal segment perfusion model for the evaluation of nutrient bioaccessibility and organ functionality ~~among~~ time for further applications to study the effect of feed ingredients and additives on nutrients bioavailability.

2 Materials and Methods

2.1 Organ harvesting and extracorporeal circulation

One gastrointestinal tract was harvested at the slaughterhouse from a 100kg Large White sow, following the Maastricht criteria for cardiac death organ donation (CDC) for transplantation purposes (15). Heterologous blood was collected at the slaughterhouse and 25 IU of heparin and 1 g of Cefazolin were added to avoid coagulation and bacterial contaminations. The blood was stored at 4 °C until organ perfusion. During the period of the “warm ischemia”, that lasted from the animal exsanguination until the duodenum isolation, the mesenteric artery was cannulated (16). The “cold ischemia” started with arterial infusion of 2 L of Belzer UW Cold Storage solution (S.A.L.F. Spa, Bergamo, Italy) (17) within 9 minutes for organ structure preservation. The descendant part of duodenum was isolated, and an intraluminal access was set-up by fixing a luer-lock connector on the pylorus side. The intestinal segment was intraluminal perfused with 360 mL of sterile Krebs-Ringer buffer (NaCl 115 mmol/L, K_2HPO_4 2.4 mmol/L, KH_2PO_4 0.4 mmol/L, $CaCl_2$ 1.2 mmol/L, $MgCl_2$ 1.2 mmol/L, $NaHCO_3^-$ 25 mmol/L, glucose 10 mmol/L) according to Biolley et al. (18) for favoriting the epithelial structure conservation. The organ was transported to the laboratory submerged in a Ringer’s lactate solution at 4°C. Duodenum tract was maintained in controlled pressure conditions (flux pump 1.48 L/min, O_2 2 L/min, artery

pressure 76/55 mmHg) through extracorporeal circulation with heterologous blood at sub-normothermic perfusion condition for 3 hours starting by the addition of warm blood at 37 °C. The circuit was composed by a peristaltic pump (as a beating heart), venous reservoir and blood oxygenator (as lungs) (19). Organ temperature was monitored using a thermometer for the entire experimental period.

2.2 Sample collection and analyses

During the first hour of extracorporeal circulation blood samples were collected from the line at 7 minutes interval (T0, T1, T2, T3, T4, T5, T6, T7, T8). Throughout the second hour, hematic samples were collected each 15 minutes (T9, T10, T11, T12) and at the third hour the collection was performed at 30 minutes intervals (T13, T14). Plasma was obtained by centrifugation at 3000 rpm for 15 min at 4 °C. In addition, the intestinal content was collected from T0 to T14 every 30 minutes from the luer-lock connector. All biological samples were evaluated for mineral and metabolites concentrations.
Glucose concentration was evaluated immediately after blood and intestinal lumen solution sampling from extracorporeal circulation line and the luer-lock connector using a glucometer (U-Right 4278, Biochemical Systems International S.p.A, Arezzo, Italy). The content of main macro-elements (Na^+, K^+, Mg^{2+}, Ca^{2+}) was assessed using Inductively Coupled Plasma Optical Emission Spectroscopy (ICP-OES, Optima 3300 XL, Perkin Elmer Inc., USA). First, calibration curves for each element considered were obtained using certified reference materials. Blood and intestinal content samples were then diluted 1:100 (v/v) with sterile MilliQ water, filtered with 0.45 um syringe filters and injected. The pH of intestinal lumen content was evaluated with pHmeter. Lactate dehydrogenase (LDH) and nitrite ion (NO_2^-) concentrations were evaluated through colorimetric kits (CytoTox 96® Non-Radioactive Cytotoxicity Assay and Griess Reagent System, Promega Italia S.r.l, Milan, Italy) following the manufacturer instructions. In particular, absorbances were red at 490 nm for LDH and 540 nm for NO_2^- using a spectrophotometer (Model 680 Microplate Reader; Bio-Rad Laboratories, Inc., Hercules, CA, USA).

2.3 Histology, immunohistochemistry, and digital image analysis

After 3 hours of continuous perfusion, the intestinal segment was subdivided in three portions (proximal A, medial B, and distal C; Figure 1) and tissue samples were fixed in 10% neutral buffered formalin, embedded in paraffin, sectioned at 4 µm, and stained with hematoxylin and eosin (H&E).
To assess the extent of epithelial cells proliferation in the intestinal mucosa, immunohistochemistry with anti-ki67 (SP6, RM-9106-S, Labvision) primary antibody was performed. Four µm sections were

deparaffinized and underwent heat-induced epitope retrieval at pH 9, for 40 min at 95°C (Dewax and HIER Buffer H, TA-999-DHBH, Thermo Scientific, UK). Endogenous peroxidase activity was blocked by incubating sections in 3% H_2O_2 for 10 min. Slides were rinsed and treated with PBS containing 10% normal serum for 30 min to reduce nonspecific background staining and then incubated for 1 hour at room temperature with the primary antibodies. Sections were incubated for 30 min with appropriate biotinylated secondary antibodies (Vector Laboratories, Burlingame, CA, USA), and then labelled by the avidin-biotin-peroxidase (ABC) procedure with a commercial immunoperoxidase kit (VECTASTAIN Elite ABC HRP Kit Standard, PK-6100, Vector Laboratories, Burlingame, CA, USA). The immunoreaction was visualized with 3,3'-diaminobenzidine substrates (Peroxidase DAB Substrate Kit, VC-SK-4100-KI01, Vector Laboratories, Burlingame, CA, USA) for 5 min and sections were counterstained with Mayer's hematoxylin. Digital slides were obtained from H&E, and immunostained sections by using the NanoZoomer-S60 Digital slide scanner (Hamamatsu, C13210-01), and images were captured by using theNDP.view2 Viewing software (Hamamatsu, U12388-01). To assess the proliferation of the intestinal mucosa, the % of ki67-positive epithelial cells (number of ki67 positive nuclei/number of total nuclei x 100) was evaluated using the ImageJ analysis program (http://rsb.info.nih.gov/ij/).

2.4 Statistical analysis

The results of blood and intestinal content glucose, minerals and metabolites were analyzed using a one-way ANOVA using JMP® Pro 15 (SAS Inst. Inc., Cary, NC, USA). Multiple comparisons among timepoints were evaluated by performing Tukey's Honestly Significant Difference test (Tukey's HSD). The results were presented as least square means ± standard errors (SE). The means were considered different when $p \leq 0.05$.

3 Results

During the extracorporeal circulation, pressure and oxygenation conditions were maintained stable showing no alterations for three hours. Organ temperature was progressively increasing over time, maintaining the sub-normothermic perfusion throughout the experimental period (T0 = 15.2 °C, T1 = 17.5 °C, T2 = 18.8 °C, T3 = 19.2 °C, T4 = 20.5 °C, T5 = 21.0 °C, T6 = 22.0 °C, T7 = 23.0 °C, T8 = 23.6 °C, T9 = 24.5 °C, T10 = 25.0 °C, T11 = 25.0 °C, T12 = 25.6 °C, T13 = 25.6 °C, T14 = 25.8 °C). In general, the intestinal segment showed redness due to blood vessels reperfusion, except in the distal portion C that appeared pale and bloodless, and the peristaltic activity was observed after pinching stimulation. Reaching 25.3 °C the peristaltic contraction was noted without any external stimulation.

3.1 Glucose, mineral concentrations, organ temperature and intestinal pH

The glucose level measured in Krebs-Ringer solution was 123.67±2.31 mg/dL. The glycemia revealed a constant decreasing trend from an initial concentration of 44.00±1.20 to 27.50±0.41 mg/dL after 3 hours of extracorporeal circulation (Figure 2).

Considering the sampling intervals, during the first hour (T0-T8) statistically significant different concentrations were registered comparing T0 to T5, T6, T7 and T8 (44.00±1.20, 36.50±1.20, 41.50±1.20, 38.25±1.20, 38.50±1.20 mg/dL respectively ($p < 0.001$). In the course of the second (T8-T12) and third hour (T12-T14) glucose concentration gradually decreased until the end of experiment ($p < 0.01$). The glucose concentrations of intestinal lumen did not significantly differ from the beginning to the end of extracorporeal circulation (T0: 94.00±6.43 mg/dL; T4: 99.50±6.43 mg/dL; T8: 97.50±6.43; T12: 90.50±4.55 mg/dL; T14: 84.00±5.25).

Considering the mineral concentrations, Ca^{2+}, Mg^{2+}, K^{+} and Na^{+} showed significant differences over the experimental time (Figure 3). In particular, Na^{+} blood levels raised from T0 to T4 and decreased slightly at T14, while its concentration in intestinal content was constantly reducing over time ($p < 0.01$). Ca^{2+}, Mg^{2+}, K^{+} blood concentrations increased significantly over time, and co-currently their concentrations in the intestinal lumen significantly dropped from T0 to T14 ($p < 0.01$).

Intestinal pH showed a constant value of 7 registered for T0, T4, T8, T12 and T14.

3.2 Lactate dehydrogenase and nitrite concentrations.

LDH blood concentrations revealed a persistent trend over time even if statistically significant differences were observed in T3 and T4 compared to T14 (T3: 0.77±0.05 OD, T4: 0.85±0.05 OD and T4: 1.08±0.05 OD; $p < 0.05$; Table 1). Intestinal lumen content registered raising concentrations of LDH from T4 to T14 ($p < 0.0001$).

Nitrite ion concentrations were below the detection level of the assay for the entire experimental period (< 1.56 µM).

3.3 Histological evaluation of the intestinal segment

In all examined portions (A, B, C), the intestinal architecture was preserved. A diffuse moderate infiltration of lymphocytes, plasma cells and lesser numbers of neutrophils was present in the intestinal mucosa, consistent with a diffuse moderate subacute enteritis. Moderate hyperemia was present in portions A and B (Figure 4). The intestinal epithelium of A and B portions were similar in terms of a

slight de-epithelialization in the apical part of villi (Figure 4A, Figure 4B). On the contrary, the intestinal portion C showed absence of erythrocytes within blood vessels (impaired perfusion) and a diffuse necrosis of villi was visible (Figure 4C). Immunohistochemistry analysis revealed 47.58% of proliferating epithelial cells in portion A, 35.01% in portion B and 33.72% in portion C (Figure 5).

4 Discussion

This study aimed to investigate the possibility to develop an alternative perfused intestinal segment model to study the nutrient bioaccessibility and bioavailability targeting to provide more complete data compared to other *in vitro* models. It is important to consider that this model is more advanced if compared to a classic *ex vivo* model which do not provide extracorporeal circulation. This model was obtained following the surgery techniques used for *in vivo* organ transplantations in order to preserve the organ viability and architecture during the entire experimental period (13).

Glucose bioaccessibility was evaluated due to its relevance *in vivo* as energetic marker derived from starch and sugar digestion. The glucose level is considered a marker of cell proliferation, viability (20, 21) and functionality for the metabolic activity (22). The initial glycemic level (44.00±1.20 mg/dL) could be considered low if compared to the normal range of swine species (from 80 to 120 mg/dL) (23, 24). The observed initial low glycaemia could be due to the animal fasting before slaughtering procedures. The glycemic trend showed a decreasing curve suggesting the utilization of glucose by erythrocytes and intestinal cells and the impossibility to store sugars in the intestinal tissue after blood perfusion. We observed a peak of glucose absorption at 7 minutes of extracorporeal circulation, and after the first hour a drop of 18% was registered. Even without registering significant differences, endoluminal content of glucose showed decreasing concentrations probably due to the low amount of glucose consumed by erythrocytes for their metabolism (25). However, the observed results suggested that glucose was transported throughout its traditional mechanism. Glucose uptake is mediated via the Sodium-Glucose Cotransporter 1 (SGLT1) localized on the enterocytes membrane and its basolateral to the blood circulation is mediated primarily by the Glucose Transporter 2 (GLUT2) (26). Glucose absorption is a complex mechanism also mediated by pancreas and renin-angiotensin-aldosterone (27). In this study, the glucose absorption could not be mediated by hormonal asset (28) since it can be fully replicated only *in vivo*. The glucose concentration used was based on the defined level for the Krebs-Ringer solution as intraluminal nutrient medium. The low intraluminal glucose concentration (29) permitted to confirm organ metabolism without registering osmotic damage (30), even if it was not possible to evaluate a kinetic curve of its uptake (14). Observed results suggest that the glucose could

be considered an interesting marker for further application of this perfusion model for the evaluation of functional feed additives which are meant to improve gut health and nutrient utilization (31, 32).

Minerals are essential inorganic nutrients that have to be exclusively acquired from the diet (33). Minerals concentration was evaluated as indirect indicator of intestinal function as they do not require digestion to be absorbed. Na^{+} concentration in blood displayed a significant increase from T0 to T7 and decreased from T7 to T14. In the meantime, the intestinal lumen content of Na^{+} dropped significantly from T0 to T14. The observed increase in osmolarity suggested that Na^{+} uptake and utilization was maintained during the extracorporeal circulation. The absorption of Na^{+} is associated with glucose uptake from Sodium-Glucose Transporters (SGLT1) (34). In this model, the physiologic separation of vascular, interstitial, and intracellular sections was conserved, and the blood concentration of Na^{+} required to maintain a correct balance of its level in the interstitial matrix.

K^{+} is one of the most important minerals for the acid-base and osmotic pressure balance (35). K^{+} is the most abundant cation ~~in the intracellular~~ and its plasma level is lower than sodium abundance (36). Similar to what observed for Na^{+}, K^{+} levels increased in blood and decreased in lumen content from T0 to T14.

Mg^{2+} is one of widely abundant minerals in the animal body, and it is involved in several pivotal processes such as energy production, muscular contraction and nervous impulses transferring (37). The observed increased Mg^{2+} plasma concentration suggests its absorption from intestinal lumen. In our study, Mg^{2+} blood level showed a drop from T0 to T7 probably due to epithelial utilization of this mineral. Subsequently, the Mg^{2+} concentration increased from T7 to T14 indicating its absorption from the lumen content. The intestinal lumen levels of Mg^{2+} significantly dropped from T0 to T14 indicating a duodenal uptake through passive and facilitated diffusion processes (38). Ca^{2+} absorption is achieved through the active transport and passive diffusion (39). Its absorption is related to the Mg^{2+} presence, involving the ATP-dependent ionic pumps that transfer Ca^{2+} in the extracellular space exchanging calcium with Na^{+} (40). Mg^{2+} could be also indirectly influenced by Na^{+} and K^{+} concentrations since it is involved in the activity of the sodium-potassium pump (41). Gastrointestinal system and kidneys closely regulate Mg^{2+} absorption and elimination (42), and its intestinal active and passive uptake mechanisms seem to do not be under hormonal control (43). For this reason, Mg^{2+} concentration could be considered a translational parameter to *in vivo,* that indicates the nutrients' bioaccessibility in the following perfusion model.

In line with the registered trend of Mg^{2+}, Ca^{2+} levels significantly raised in blood and dropped in the intestinal lumen content. Ca^{2+}, Mg^{2+} and K^{+} plasma concentrations showed a decrease in case of enteritis *in vivo* which can impair the absorption functionality of the gut epithelium (44, 45). In our

duodenum perfusion segment model, luminal and plasma mineral concentrations demonstrated that the bioaccessibility of these nutrients was maintained over three hours of extracorporeal circulation.

The lactate dehydrogenase is a cytosolic enzyme that catalyzes the conversion of lactate to pyruvate. LDH measurements offer a non-invasive and objective indication of mucosal cellular integrity since the extracellular localization of LDH is associated with an epithelial tissue injury (46). LDH blood concentrations did not show any difference over time even if higher numerical values were registered after 3 hours. During an organ injury LDH is released from dead cells and its concentration increases both in plasma and intestinal content (47). In addition, LDH is strongly correlated with hemoglobin release in blood vessels due to hemolysis (48). The registered levels of LDH in plasma suggests that peristaltic pump and pressure conditions used for extracorporeal circulation were suitable in terms of erythrocytes integrity (49). The perfused intestinal segment model showed a progressive increase of luminal concentration of LDH suggesting a progressive damage of epithelial mucosa, consistent with the intestinal de-epithelialization and necrosis observed during the histological evaluation.

In this model, LDH concentrations in plasma and intestinal lumen content could be considered as reliable indirect ~~control~~ to assess organ viability over time.

In line with Sundaram et al. (50), nitric oxide was not detectable, probably due to the limited ability of swine enterocytes to produce NO_2^-.

The preservation of tissue integrity before and during the experimentation is pivotal to ensure the accuracy and reproducibility of data (51). Histological evaluation revealed that the overall intestinal architecture was preserved after 3 hours, with a visible blood perfusion and only a slight de-epithelialization of villi in portions A and B, while in portion C perfusion was absent and a diffuse loss of intestinal villi was observed. In this distal portion the nutrient absorption was likely impaired. Intestinal proliferation ~~resulted~~ highest in proximal portion A with a progressive decrease in B and C, supporting a reduced vitality in particular in the distal portion C. In the examined portions of duodenum, the observed enteritis was considered a spontaneous finding not unexpected in a farming pig. Preservation of epithelial structures for 3 hours was proposed as optimal period to optimize organ viability in *ex vivo* studies (52). The observed increase in LDH was likely due to the de-epithelization of the intestinal mucosa. Hyperemia observed in the portions A and B was probably due to high pressure conditions, even if the selected parameters were in accordance with previous studies. In this model, pressure conditions require to be adapted in accordance with the size of the considered intestinal segment, and this aspect will require further adaptations in order to perfection this model. Literature studies demonstrated that UW solution provides better conservation of patches through prolonged ischemia compared to other solutions (53). Further studies are needed to assess the glucose absorption

trough perfusion of higher glucose concentrations through the intraluminal line. In addition, more data are required to investigate and optimize the luminal nutrition in order to maximize the organ viability and the conservation of intestinal structure for longer periods.

5 Conclusions

The developed swine perfused intestinal segment model showed characteristics of organ viability and functionality over three hours of extracorporeal circulation. The duodenum segment preserved the principal nutrients bioaccessibility, cellular metabolism, and, at histological evaluation, showed a preserved intestinal epithelial lining in the perfused portions. This study offered encouraging results for the development of a novel swine intestinal segment perfusion model for the evaluation of nutrients bioaccessibility in line with the 3R principle. Future studies will be useful to improve the organ viability and structures conservation, considering the potential of this model also for application to translational medicine for intestinal transplantations.

Author Contributions

Conceptualization: Luciana Rossi, Fabio Acocella. **Methodology**: Matteo Dell’Anno, Elisabetta Bongiorno, Camilla Recordati, Pietro Riccaboni, Fabio Acocella, Luciana Rossi. **Formal analysis**: Matteo Dell’Anno, Elisabetta Bongiorno, Pietro Riccaboni, Camilla Recordati, Fabio Acocella, Luciana Rossi. **Investigation**: Fabio Acocella, Matteo Dell’Anno, Luciana Rossi, Pietro Riccaboni, Camilla Recordati, Elisabetta Bongiorno. **Data curation**: Matteo Dell’Anno. **Writing-original draft preparation**: Matteo Dell’Anno, Fabio Acocella. **Review and editing**: Luciana Rossi, Fabio Acocella, Camilla Recordati, Pietro Riccaboni, Matteo Dell’Anno. **Visualization**: Matteo Dell’Anno, Pietro Riccaboni, Camilla Recordati. **Supervision**: Luciana Rossi, Fabio Acocella. **Funding acquisition**: Luciana Rossi.
All authors drafted and approved the final version of the manuscript.

Financial Disclosure Statement

This research was funded by Lombardy Region (funding number: POR FESR 2014–2020_BANDO Call HUB Ricerca e Innovazione_D.G.R. NR 727 del 5 November 2018) within the project MIND FoodS Hub (Milano Innovation District Food System Hub): Innovative concept for the eco-intensification of agricultural production and for the promotion of dietary patterns for human health and longevity through the creation in MIND of a digital Food System Hub. The funder has no role in defining the study, in data collection, management, analysis or interpretation of the data, nor in the writing and submitting of manuscripts resulting from this study.

Declaration of Competing Interest

The authors declare that the research was conducted in the absence of any commercial or financial relationships that could be construed as a potential conflict of interest.

Data Statement

All data are available within the manuscript.

References

1. Ross AC, Caballero B, Cousins RJ, Tucker KL, Ziegler TR. Modern nutrition in health and disease: Lippincott Williams & Wilkins; 2012.
2. Domínguez Díaz L, Fernández-Ruiz V, Cámara M. The frontier between nutrition and pharma: The international regulatory framework of functional foods, food supplements and nutraceuticals. Critical reviews in food science and nutrition. 2020;60(10):1738-46.
3. Novik G, Savich V. Beneficial microbiota. Probiotics and pharmaceutical products in functional nutrition and medicine. Microbes and Infection. 2020;22(1):8-18.
4. Hull MA. Nutritional prevention of colorectal cancer. Proceedings of the Nutrition Society. 2021;80(1):59-64.
5. Devle H, Ulleberg EK, Naess-Andresen CF, Rukke E-O, Vegarud G, Ekeberg D. Reciprocal interacting effects of proteins and lipids during ex vivo digestion of bovine milk. International Dairy Journal. 2014;36(1):6-13.
6. Jin IS, Yoon MS, Park C-W, Hong JT, Chung YB, Kim J-S, et al. Replacement techniques to reduce animal experiments in drug and nanoparticle development. Journal of Pharmaceutical Investigation. 2020;50(3):327-35.
7. Griffiths NM, Van der Meeren A, Angulo JF, Vincent-Naulleau S. Research on the Radiotoxicology of Plutonium Using Animals: Consideration of the 3Rs—: Replace, Reduce, Refine. Health physics. 2020;119(1):133-40.
8. Blume C, Davies DE. In vitro and ex vivo models of human asthma. European Journal of Pharmaceutics and Biopharmaceutics. 2013;84(2):394-400.
9. Lueangsakulthai J, Sah B, Scottoline B, Dallas D. Survival of Recombinant Monoclonal Antibodies (IgG, IgA and sIgA) Versus Naturally-Occurring Antibodies (IgG and sIgA/IgA) in an Ex Vivo Infant Digestion Model. Nutrients. 2020;12(3).
10. Baydoun M, Treizeibre A, Follet J, Vanneste S, Creusy C, Dercourt L, et al. An Interphase Microfluidic Culture System for the Study of Ex Vivo Intestinal Tissue. Micromachines. 2020;11(2).
11. Salehi P, Zhu JZ, Castillo EG, Avila J, Lakey J, Churchill TA. Preserving the mucosal barrier during small bowel storage1. Transplantation. 2003;76(6):911-7.
12. Ziegeler S, Raddatz A, Hoff G, Buchinger H, Bauer I, Stockhausen A, et al. Antibiotics modulate the stimulated cytokine response to endotoxin in a human ex vivo, in vitro model. Acta anaesthesiologica scandinavica. 2006;50(9):1103-10.
13. Stangl M, Krapp J, Theodorou D, Eder M, Hammer C, Land W, et al. Computer-assisted ex vivo, normothermic small bowel perfusion. European surgical research. 2000;32(2):100-6.
14. Hamed M, Barlow A, Dolezalova N, Khosla S, Sagar A, Gribble F, et al. Ex vivo normothermic perfusion of isolated segmental porcine bowel: a novel functional model of the small intestine. BJS open. 2021;5(2):zrab009.

15. Thuong M, Ruiz A, Evrard P, Kuiper M, Boffa C, Akhtar MZ, et al. New classification of donation after circulatory death donors definitions and terminology. Transplant International. 2016;29(7):749-59.
16. Lauronen J, Pakarinen MP, Pirinen P, Kuusanmãki P, Raivio P, Savilahti E, et al. Effects of extrinsic denervation with or without ischemia–reperfusion injury on constitutional mucosal characteristics in porcine jejunoileum. Digestive diseases and sciences. 2001;46(3):476-85.
17. Guibert EE, Petrenko AY, Balaban CL, Somov AY, Rodriguez JV, Fuller BJ. Organ preservation: current concepts and new strategies for the next decade. Transfusion Medicine and Hemotherapy. 2011;38(2):125-42.
18. Biolley C, Tretola M, Bee G, Jud C, Silacci P. Punicalagin increases glutamate absorption in differentiated Caco-2 cells by a mechanism involving gene expression regulation of an EAAT3 transporter. Food & function. 2019;10(9):5333-8.
19. Alexandre F, Fabiani J. Circolazione extracorporea. EMC-Tecniche Chirurgiche-Chirurgia Generale. 2010;10(1):1-22.
20. Li Y, Schilling T, Benisch P, Zeck S, Meissner-Weigl J, Schneider D, et al. Effects of high glucose on mesenchymal stem cell proliferation and differentiation. Biochemical and Biophysical Research Communications. 2007;363(1):209-15.
21. MacIver NJ, Jacobs SR, Wieman HL, Wofford JA, Coloff JL, Rathmell JC. Glucose metabolism in lymphocytes is a regulated process with significant effects on immune cell function and survival. Journal of leukocyte biology. 2008;84(4):949-57.
22. Clara R, Schumacher M, Ramachandran D, Fedele S, Krieger JP, Langhans W, et al. Metabolic adaptation of the small intestine to short- and medium- term high- fat diet exposure. Journal of cellular physiology. 2017;232(1):167-75.
23. Uc A, Olivier AK, Griffin MA, Meyerholz DK, Yao J, Abu-El-Haija M, et al. Glycaemic regulation and insulin secretion are abnormal in cystic fibrosis pigs despite sparing of islet cell mass. Clinical science. 2015;128(2):131-42.
24. Lombardi P, Musco N, Calabrò S, Tudisco R, Mastellone V, Vastolo A, et al. Different carbohydrate sources affect swine performance and post-prandial glycaemic response. Italian Journal of Animal Science. 2020;19(1):421-30.
25. Nossol C, Barta-Böszörményi A, Kahlert S, Zuschratter W, Faber-Zuschratter H, Reinhardt N, et al. Comparing two intestinal porcine epithelial cell lines (IPECs): morphological differentiation, function and metabolism. PloS one. 2015;10(7):e0132323.
26. Croset M, Rajas F, Zitoun C, Hurot J-M, Montano S, Mithieux G. Rat small intestine is an insulin-sensitive gluconeogenic organ. Diabetes. 2001;50(4):740-6.
27. Giacchetti G, Sechi LA, Rilli S, Carey RM. The renin–angiotensin–aldosterone system, glucose metabolism and diabetes. Trends in Endocrinology & Metabolism. 2005;16(3):120-6.
28. Khan A, Pessin J. Insulin regulation of glucose uptake: a complex interplay of intracellular signalling pathways. Diabetologia. 2002;45(11):1475-83.
29. Verhoeckx K, Cotter P, López-Expósito I, Kleiveland C, Lea T, Mackie A, et al. The impact of food bio-actives on gut health. Vitro and ex vivo models. 2015:83-93.
30. Quan G-B, Han Y, Yang C, Hu W-B, Liu M-X, Liu A, et al. Inhibitory effect of trehalose on phosphatidylserine exposure, osmotic fragility and membrane lipid peroxidation damage of erythrocytes induced by high concentration of glucose. Zhongguo shi yan xue ye xue za zhi. 2008;16(6):1442-6.
31. Sotira S, Dell'Anno M, Caprarulo V, Hejna M, Pirrone F, Callegari ML, et al. Effects of Tributyrin Supplementation on Growth Performance, Insulin, Blood Metabolites and Gut Microbiota in Weaned Piglets. Animals. 2020;10(4):726.

32. Miragoli F, Patrone V, Prandini A, Sigolo S, Dell'Anno M, Rossi L, et al. Implications of Tributyrin on Gut Microbiota Shifts Related to Performances of Weaning Piglets. Microorganisms. 2021;9(3):584.
33. Samat NA, Yusoff FM, Rasdi NW, Karim M. Enhancement of live food nutritional status with essential nutrients for improving aquatic animal health: A review. Animals. 2020;10(12):2457.
34. Navale AM, Paranjape AN. Glucose transporters: physiological and pathological roles. Biophysical reviews. 2016;8(1):5-9.
35. Baloš MŽ, Jakšić S, Knežević S, Kapetanov M. Electrolytes–sodium, potassium and chlorides in poultry nutrition. Archives of Veterinary Medicine. 2016;9(1):31-42.
36. Udensi UK, Tchounwou PB. Potassium homeostasis, oxidative stress, and human disease. International journal of clinical and experimental physiology. 2017;4(3):111.
37. Gröber U, Schmidt J, Kisters K. Magnesium in prevention and therapy. Nutrients. 2015;7(9):8199-226.
38. Scillitani A, Pugliese F, Salcuni AS, Carnevale V. Regolazione del metabolismo del calcio, del fosfato e del magnesio. L'Endocrinologo. 2020;21(5):348-53.
39. Duan P, Yuan X, Gan S, Zhang Y, Duan W. Time delay induces saw-tooth calcium wave and transition in intracellular calcium oscillation. Chinese Journal of Physics. 2020;66:150-6.
40. Veklich T, IuIu M, Kosterin S. Mg2+, ATP-dependent plasma membrane calcium pump of smooth muscle cells. I. Structural organization and properties. Ukrainian biochemical journal. 2015;87(1):5-20.
41. Rodrigues AM, Silva DB, Miranda MF, Braga da Silva SC, Canton Santos LE, Scorza FA, et al. The Effect of Low Magnesium Concentration on Ictal Discharges In A Non-Synaptic Model. International Journal of Neural Systems. 2021;31(04):2050070.
42. Al Alawi AM, Al Badi A, Al Huraizi A, Falhammar H. Magnesium: The recent research and developments. Advances in Food and Nutrition Research. 2021;96:193-218.
43. Vormann J. Magnesium: nutrition and metabolism. Molecular aspects of medicine. 2003;24(1-3):27-37.
44. Bhat AA, Wadhwa DR, Singh SP, Singh I. Haematological and biochemical analysis in canine enteritis. Vet World. 2013;6(7):380-3.
45. Kimmel SE, Waddell LS, Michel KE. Hypomagnesemia and hypocalcemia associated with protein-losing enteropathy in Yorkshire terriers: five cases (1992–1998). Journal of the American Veterinary Medical Association. 2000;217(5):703-6.
46. Sreenivasan PK, Kakarla VVP, Sharda S, Setty Y. The effects of a novel herbal toothpaste on salivary lactate dehydrogenase as a measure of cellular integrity. Clinical oral investigations. 2021;25(5):3021-30.
47. Mura M, Andrade CF, Han B, Seth R, Zhang Y, Bai X-H, et al. Intestinal ischemia-reperfusion-induced acute lung injury and oncotic cell death in multiple organs. Shock. 2007;28(2):227-38.
48. Sullivan I, Faulds J. Lactate dehydrogenase and Haemolysis Index as quality control markers of haemolysis in intra- operative cell salvage. Transfusion medicine. 2013;23(5):326-9.
49. Poder T, Boileau JC, Lafrenière R, Thibault L, Carrier N, de Grandmont MJ, et al. Quantitative assessment of haemolysis secondary to modern infusion pumps. Vox sanguinis. 2017;112(3):201-9.
50. Sundaram TS, Giromini C, Rebucci R, Baldi A. Omega-3 polyunsaturated fatty acids counteract inflammatory and oxidative damage of non-transformed porcine enterocytes. Animals. 2020;10(6):956.
51. Sjöberg Å, Lutz M, Tannergren C, Wingolf C, Borde A, Ungell A-L. Comprehensive study on regional human intestinal permeability and prediction of fraction absorbed of drugs using the Ussing chamber technique. European Journal of Pharmaceutical Sciences. 2013;48(1-2):166-80.

52. Sutton ME, op den Dries S, Karimian N, Weeder PD, de Boer MT, Wiersema-Buist J, et al. Criteria for viability assessment of discarded human donor livers during ex vivo normothermic machine perfusion. PloS one. 2014;9(11):e110642.
53. de Sousa SG, Nascimento da Silva GV, AM CR, da Silva MF, Costa FC, Santana de Macedo B, et al. Organ Preservation Solutions in Transplantation: A Literature Review. Experimental and Clinical Transplantation: Official Journal of the Middle East Society for Organ Transplantation. 2021.

Tables

Table 1. Lactate dehydrogenase concentrations measured over time in blood and intestinal lumen content.

Timepoint	Blood (OD)	Lumen (OD)
T0	0.93 ± 0.05^{ab}	0.32 ± 0.02^{a}
T1	0.99 ± 0.05^{ab}	
T2	0.81 ± 0.05^{ab}	
T3	0.77 ± 0.05^{a}	
T4	0.84 ± 0.05^{a}	0.34 ± 0.02^{a}
T5	0.87 ± 0.05^{ab}	
T6	0.82 ± 0.05^{ab}	
T7	0.89 ± 0.05^{ab}	
T8	0.89 ± 0.05^{ab}	0.50 ± 0.02^{b}
T9	0.86 ± 0.05^{ab}	
T10	0.92 ± 0.05^{ab}	
T11	0.83 ± 0.05^{ab}	
T12	0.92 ± 0.05^{ab}	0.80 ± 0.02^{c}
T13	1.01 ± 0.05^{ab}	
T14	1.08 ± 0.05^{b}	1.36 ± 0.02^{d}
p-value	0.0256	< 0.0001

Data are expressed as least squared means and standard errors.

Different lowercase letters indicate statistically significant differences ($p \leq 0.05$).

OD: optical densities measured at 490 nm of wavelength.

Figures

Figure 1. Representative image of the intestinal segment perfusion model, ideally subdivided in proximal (A), medial (B) and distal (C) portions for tissue sampling. The outlined lines indicate the incision points.

Figure 2. Glycemic levels measured for 3 hours from the extracorporeal circulation of perfused intestinal segment.

Data are expressed as least squared means and standard errors.

Different lowercase letters indicate statistically significant differences ($p \leq 0.01$).

First hour: hematic glucose concentrations of the first hour measured at 7 minutes intervals; Second hour: hematic glucose concentrations of the second hour measured at 30 minutes intervals; Third hour: hematic glucose concentrations of the third hour measured at 30 minutes intervals. Asterisk indicates statistically significant different values.

Figure 3. Mineral concentrations measured over time in blood plasma and intestinal lumen content.

Data are expressed as least squared means and standard errors.

Different lowercase letters indicate statistically significant differences ($p \leq 0.05$).

a) Calcium (Ca^{2+}) concentrations measured at T0-T7-T14 in blood and T0-T14 in intestinal lumen content; b) Magnesium (Mg^{2+}) concentrations measured at T0-T7-T14 in blood and T0-T14 in intestinal lumen content; c) Potassium (K^{+}) concentrations measured at T0-T7-T14 in blood and T0-T14 in intestinal lumen content; d) Sodium (Na^{+}) concentrations measured at T0-T7-T14 in blood and T0-T14 in intestinal lumen content.

Figure 4. Histology of the perfused intestinal tract at the end of the 3 hours of extracorporeal circulation (H&E stain, 100x, scale bar = 200 µm). Portions A and B were overall well preserved and perfused, as demonstrated by blood vessels engorged with erythrocytes (arrowheads). In portion C, blood vessels are not evident (impaired perfusion) and necrosis of villi (*) was present.

Hematoxylin eosin of intestinal A, B and C portions.

Figure 5. Proliferation of intestinal crypt epithelial cells.

Immunoperoxidase staining for ki67, 400x, scale bar = 20 μm in portions A, B, and C.

Figure1

Click here to access/download;Figure;Figure 1 (500 dpi).tif

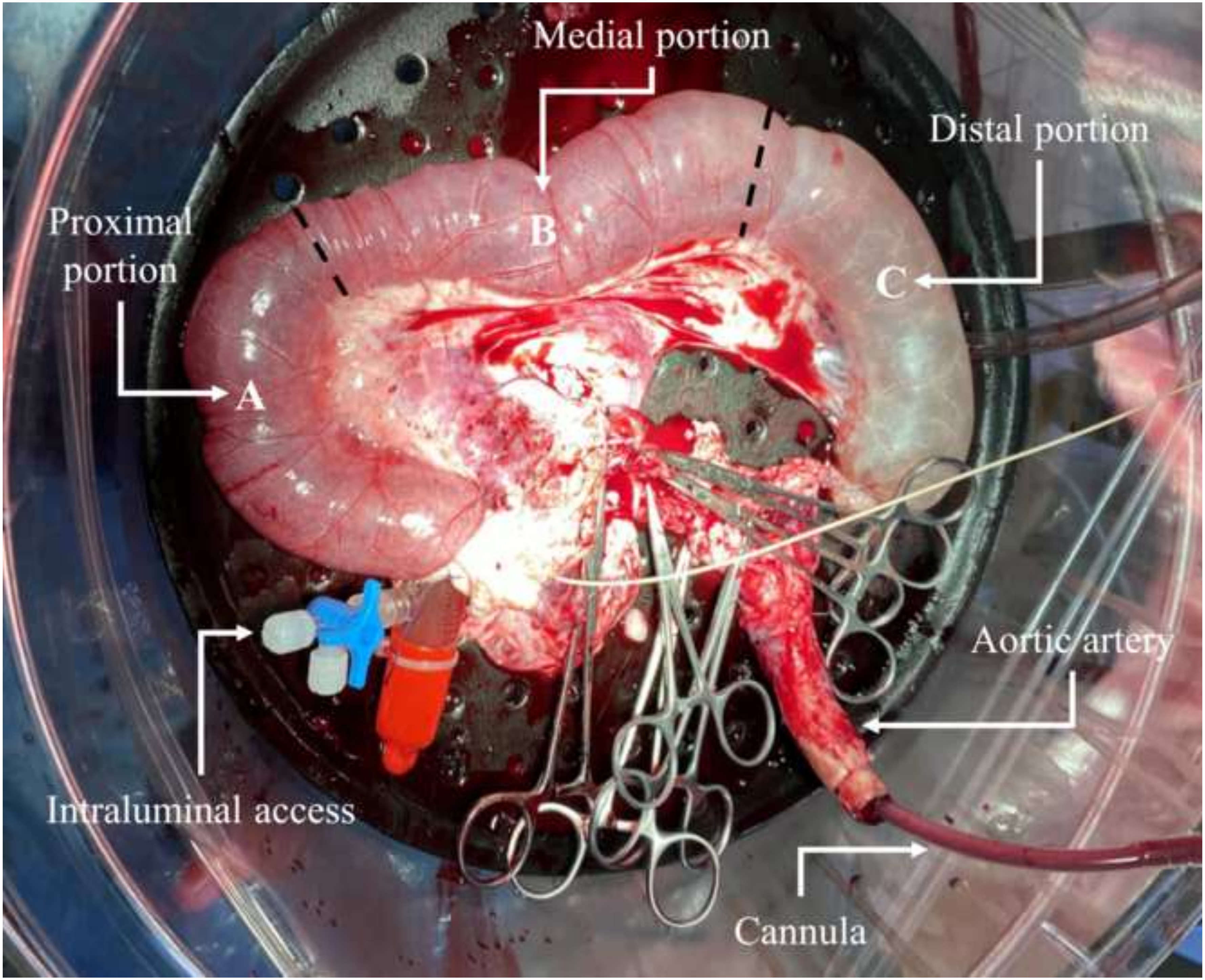

Figure 1.tif

Figure2

Click here to access/download;Figure;Figure 2 (500 dpi).tif

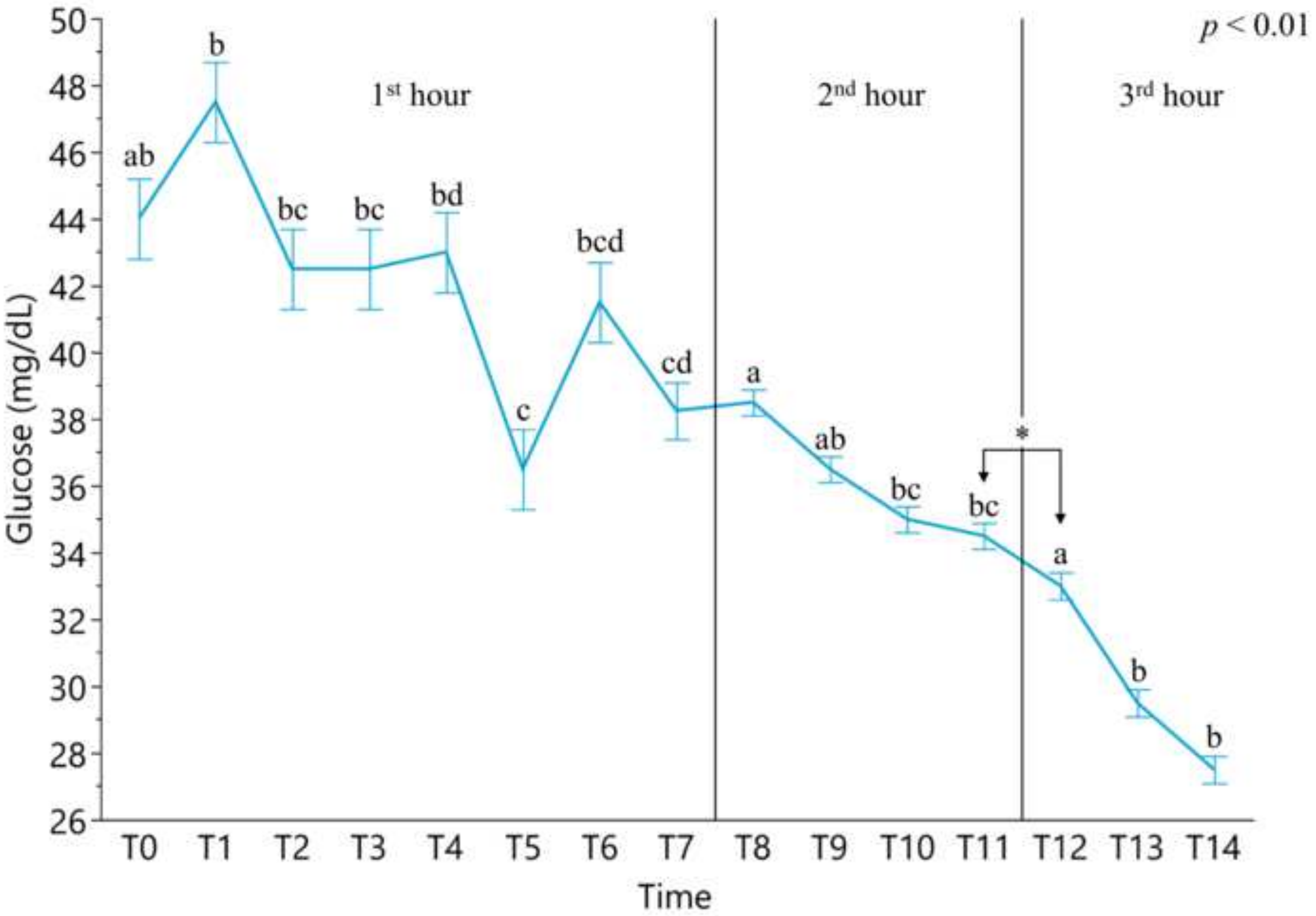

Figure 2.tif

Figure3

Click here to access/download;Figure;Figure 3 (500 dpi).tif

Figure 3.tif

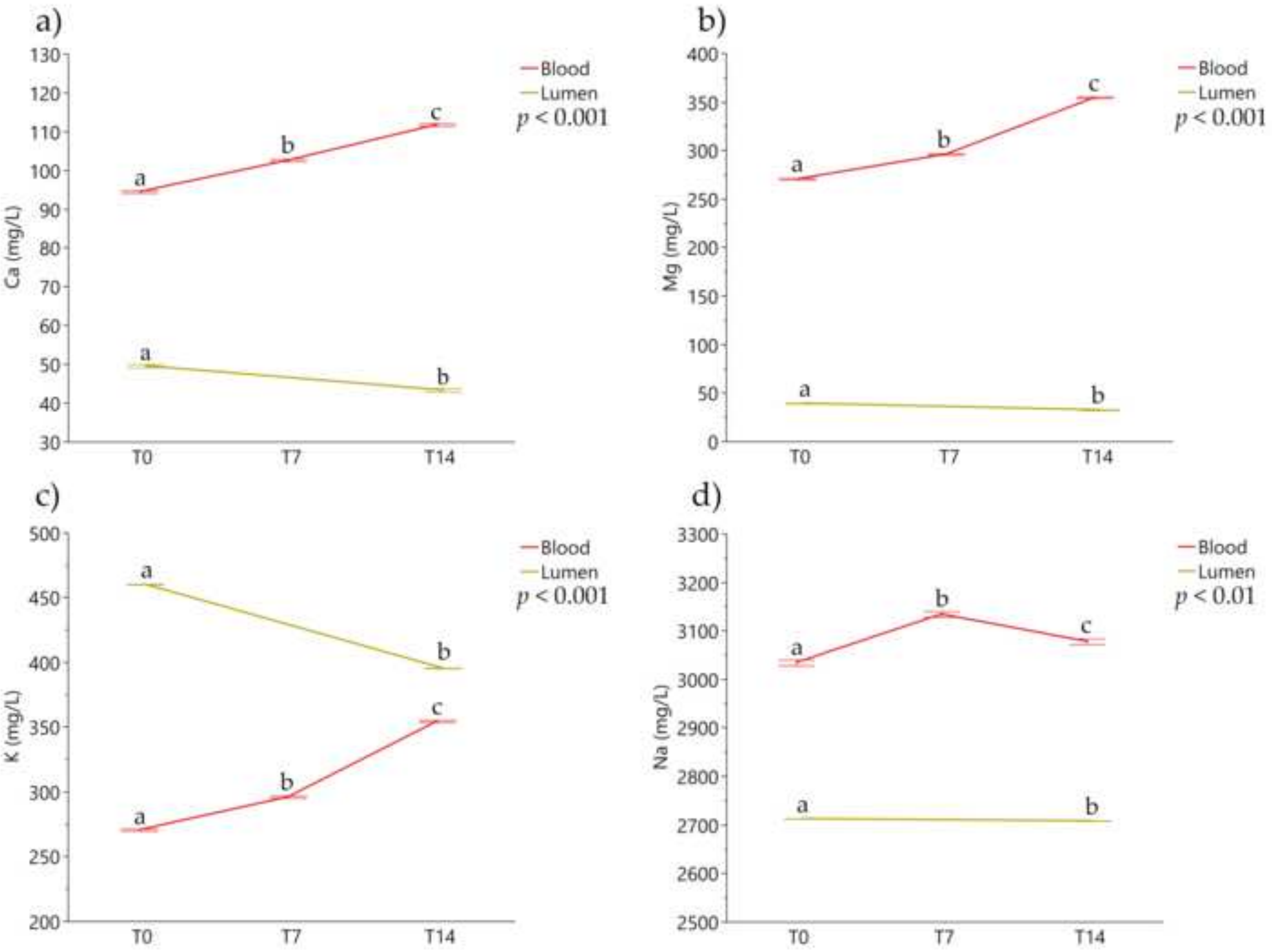

Click here to access/download;Figure;Figure 4.tif

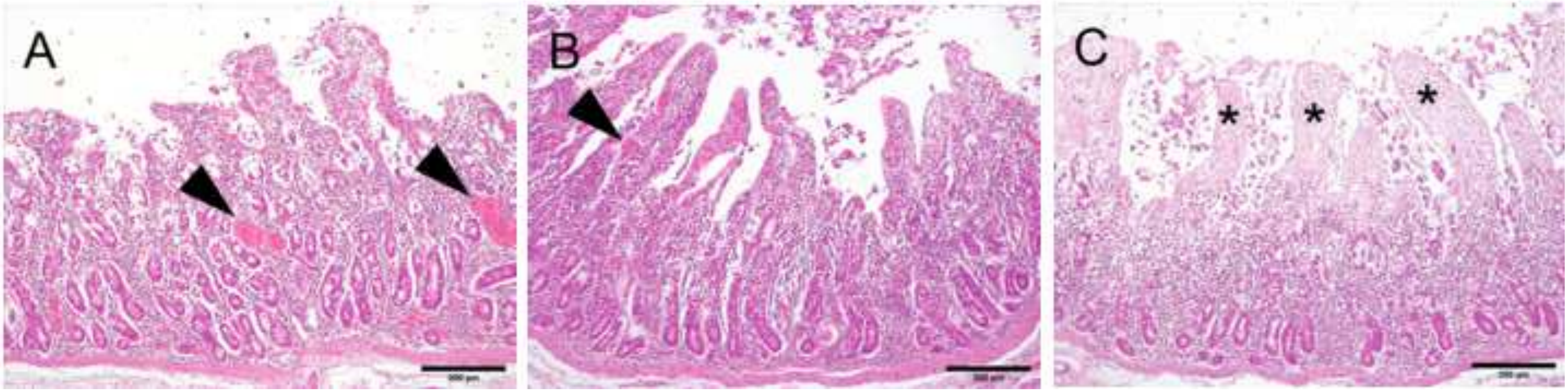

Figure5

Click here to access/download;Figure;Figure 5.tif

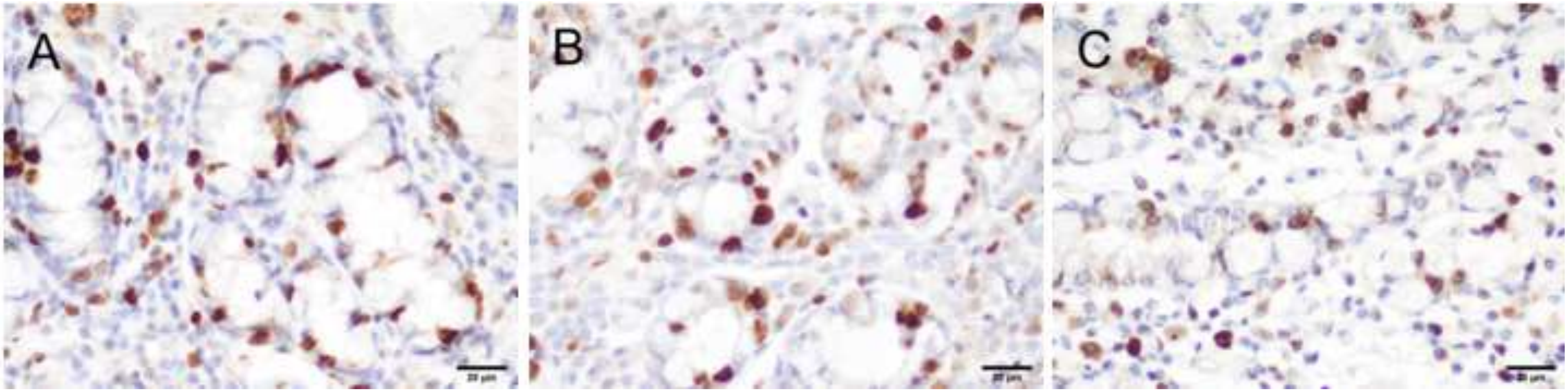

Supplement: Attachment — Submitted filename: PONE-D-22-35014.pdf [file pone.0283825.s001.pdf]
